# Changes in HER2low and HER2-ultralow status in 47 advanced breast carcinoma core biopsies, matching surgical specimens, and their distant metastases assessed by conventional light microscopy, digital pathology, and artificial intelligence

**DOI:** 10.1007/s10549-025-07776-6

**Published:** 2025-07-22

**Authors:** Anikó Kovács, Leif Klint, Barbro Linderholm, Toshima Z. Parris

**Affiliations:** 1 Department of Clinical Pathology, Region Västra Götaland, Sahlgrenska University Hospital, Gothenburg, Sweden; 2 Institute of Biomedicine, Sahlgrenska Academy, University of Gothenburg, Gothenburg, Sweden; 3 Department of Oncology, Institute of Clinical Sciences, Sahlgrenska Academy, University of Gothenburg, SE-413 45, Gothenburg, Sweden; 4 Department of Oncology, Region Västra Götaland, Sahlgrenska University Hospital, Gothenburg, Sweden; 5 Sahlgrenska Center for Cancer Research, Sahlgrenska Academy, University of Gothenburg, Gothenburg, Sweden

**Keywords:** HER2-Low breast cancer, HER2-Ultralow breast cancer, HER2-Null breast cancer, Artificial Intelligence, Changes in HER2-Low status

## Abstract

**Background:**

HER2-targeted therapies have improved survival in HER2-positive breast cancer, and recent data suggest potential benefits for patients with HER2-low tumors (defined as immunohistochemistry (IHC) 1 + or 2 + and, in situ hybridization (ISH)-negative). HER2-low tumors are heterogenous, spanning the hormone receptor-positive and triple-negative subtypes. Assessing HER2-low and HER2-ultralow status remains challenging, especially across specimen types.

**Aims:**

This study aims to (1) compare HER2 assessment using conventional microscopy, digital pathology, and an artificial intelligence (AI) model, and (2) investigate changes in HER2-low status between core biopsies, surgical specimens, and metastases.

**Materials and methods:**

IHC slides from 47 HER2-low advanced breast carcinomas were analyzed using conventional microscopy, digital pathology, and an AI model developed on Aiforia® Create. HER2 statuses were categorized as low, ultralow (score 1 + in 1–10%), and null (score 0 or 1 + in < 1% with difficult-to-interpret minimal membranous-like staining). Changes in HER2 expression across specimen types were evaluated using agreement measures and visualization tools.

**Results:**

The AI model identified more HER2-low and HER2-ultralow cases compared to conventional methods, improving detection accuracy. HER2 expression differed between specimen types, with metastases exhibiting increased HER2 expression compared to surgical specimens and core biopsies. Digital pathology also showed stronger membranous staining and identified more HER2 expressor tumor cells with any kind of membranous staining than microscopy.

**Conclusions:**

AI evaluation is a more sensitive method for HER2-low assessment and reveals expression changes across disease progression. These findings emphasize the need for standardized HER2 assessment to ensure accurate therapy eligibility, particularly for novel treatments like Trastuzumab–Deruxtecan.

**Supplementary Information:**

The online version contains supplementary material available at 10.1007/s10549-025-07776-6.

## Introduction

The availability of HER2-targeted therapies over the past two decades has significantly improved clinical outcomes for patients with HER2-positive breast cancer (BC) [1]. In this context, HER2-positive BC refers to tumors with high levels of HER2 protein expression, as assessed by immunohistochemistry (IHC), and/or HER2 amplification, as assessed by in situ hybridization (ISH) [2]. HER2-low is a new term to describe patient tumors with low HER2 expression and is defined as IHC 1 + or IHC 2 + and ISH-negative. BC patients classified as HER2-low represent a heterogeneous population, including luminal hormone receptor-positive BC and triple-negative BC [3]. According to the current HER2 testing guidelines, BC patients with HER2-low disease are categorized as having HER2-negative BC. As traditional HER2-targeted therapies have not shown efficacy in this subpopulation, these therapies are not recommended in clinical guidelines [4].

Trastuzumab deruxtecan (T-Dxd) is a novel HER2-targeted antibody–drug conjugate (ADC) developed to deliver a potent topoisomerase I inhibitor drug to HER2-expressing cancer cells with potentially reduced systemic toxicity. The drug is conjugated to a humanized anti-HER2 antibody via a cleavable, plasma-stable, peptide-based linker. Upon cleavage of the linker by lysosomal cathepsins, which are upregulated in cancer cells, the released drug becomes cell membrane-permeable. In preclinical studies, T-Dxd demonstrated antitumor activity in various tumor types, including those with low HER2 expression [5]. The antitumor effect of T-Dxd in heterogeneous or HER2-low tumors may be related to the bystander effect, the released drug can act on all nearby tumor cells [6]. This effect, combined with the high drug-to-antibody ratio (payload) and the high potency of the drug ensures high cytotoxicity at the tumor site. Two randomized phase 3 studies (DESTINY-Breast04 & DESTINY-Breast06) involving patients with metastatic BC defined as HER2-low showed that T*-*Dxd was more effective than chemotherapy in prolonging progression free survival [7, 8]. In addition, in the DESTINY-Breast06 study, around 17% of patients were classified as having so-called HER2ultra-low expression, representing a HER2-low disease with faint, incomplete membrane staining of 1 + in ≤ 10% of tumor cells.

It is important to evaluate how HER2 expression changes in the metastatic setting to assess the need for additional biopsies and to guide treatment decisions with new therapies like T-Dxd. However, assessing HER2-low status by IHC is challenging and further confirmation with molecular assays is currently not available [9, 10]. In most cases, IHC 1 + and 2 + regions are heterogeneously distributed within invasive breast carcinomas and distant metastases. Assessment is particularly difficult when the numbers of tumor cells is near the 10% cutoff point (for HER2-low versus HER2-ultralow status) or the 1% cutoff point (for HER2-ultralow versus HER2-null status) [11]. This sparked our interest in investigating whether modern techniques, such as artificial intelligence (AI), could be used as a tool to better define HER2 expression according to these subgroups compared to conventional methods, such as conventional microscopy, as well as digital pathology.

This study aimed to (1) compare the assessment and outcome of HER2status using conventional microscopy, digital pathology, and AI; and (2) assess changes in HER2-low status in core biopsies, matching surgical specimens, and distant metastases.

## Materials and methods

### Patient samples

For invasive breast carcinomas with HER2-low status (*n* = 47), IHC HercepTest slides for core needle biopsies as well as, matching surgical specimens, and distant metastases were reevaluated using three modalities: (1) Conventional microscopy (eyeballing in a light microscope), (2) Visual estimation of the scanned digital image on the screen (eyeballing on the screen), and (3) evaluation with an AI model (Aiforia®) providing an exact percentage for the HER2 score. AI analysis was preceded by deep learning using the pathologist’s (A.K.) annotations of the IHC images, with assistance from Aiforia® staff.

The original IHC HercepTest slides (stained routinely between 2013 and 2023 at the Department of Clinical Pathology, Sahlgrenska University Hospital, Gothenburg, Sweden) were retrieved from the archives. The HercepTest slides were not restained. In brief, 4 µm sections were prepared from formalin-fixed paraffin-embedded blocks. After pretreatment using the Dako PTLink system (Dako, Carpinteria, CA, USA), the sections were processed further on an automated DAKO Autostainer platform with HercepTest (Dako, Cat. SK001). For samples with HercepTest scores of 2 + , an additional Ventana dual silver in situ hybridization (SISH) test was performed.

The original HercepTest scoring was performed by six board-certified pathologists. Int the present study, reevaluation of HercepTest scoring was done by one of these pathologists (A.K.). The revised scores were assigned based on the scanned full-faced slides or the digital images. According to the HER2-low concept, the HercepTest scores were 0, 1 + and 2 + without consideration of the staining intensity. No HER2-positive or HER2-amplified cases were included in the study. A three-tiered classification system was then used to determine HER2 status using scores of 2 + , 1 + , and 0. HER2 statuses were categorized as low (scores 2 + and 1 + without amplification of the HER2 gene), ultralow (score 1 + in 1–10%), and null (only score 0 or score 1 + in < 1% with difficult-to-interpret minimal membranous-like staining). Specifically, the HER2-ultralow and HER2null categories in our study slightly differed from those defined in the CAP guidelines, as we applied a strict 1% cutoff when assessing HER2null status. Cases in which HER2 membrane staining was observed in less than 1% of tumor cells and was difficult to interpret were classified as HER2null.The original HercepTest slides were first scanned using a NanoZoomer S210/Hamamatsu scanner (Oncotopix® Scan by Visiopharm) at 40 × magnification, and subsequently uploaded to the Aiforia® website.

**Fig. 1:**
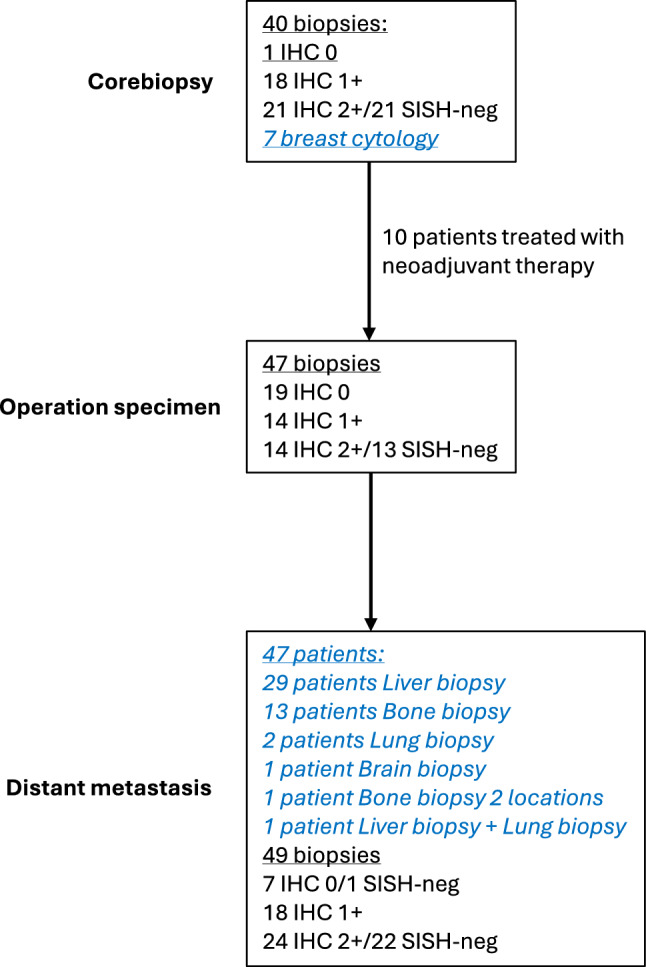
Patient characteristics. The figure shows the number of samples and their results from the three different sites

In total, 400 estimations and assessments were done in this study (Fig. 1):

- 40 core biopsies with 3 modalities = 120 examinations (7 cases were diagnosed by cytology)
- 47 surgical specimens with 3 modalities = 141 examinations
- 47 distant metastases with 3 modalities = 139 examinations (one metastasis had only a scanned image, here only eye balling on the screen was possible).

### AI model description and development

Our HER2-low model was developed utilizing whole-slide images (WSI) on a cloud-based, supervised deep-learning AI-platform—Aiforia® Create 6.0 (Aiforia Technologies Plc, Helsinki, Finland). The AI model was trained as a set of individual convolutional neural networks (CNNs), called “layers”. Layers were trained independently of each other but built in a “layer-tree”—in a “parent–child” fashion, where each “child” layer was exclusively trained on the areas identified by the “parent” layer. Each individual layer was designed to identify a single or multiple classes. Annotations were provided as training data for each class within a given layer. Our HER2-low model consisted of three layers, with each layer having a specific set of classes (total of six; Table 1).

**Table 1:** Her2 Low model layers and classes. RL – region layer; OL – object layer; FoV – field of view; VC – very complex

AI model Layer	AI model class	Morphological feature detected	Output	FoV (µm)	Complexity
RL 1	Tissue	Total breast tissue	Tissue area (mm^2^)	100	VC
RL 2	Invasive Epithelium	Malignant epithelial cells infiltrating the surrounding breast tissue	Invasive epithelium area (mm^2^)	50	VC
OL 1	Her 2_score 0	Malignant epithelial cells without Her2 staining	Count	–	VC
	Her 2_score 1 +	Malignant epithelial cells with faint incomplete Her2 staining	Count	–	VC
	Her 2_score 2 +	Malignant epithelial cells with weak to moderate complete Her2 staining	Count	–	VC
	Her 2_score 3 +	Malignant epithelial cells with strong complete Her2 staining	Count	–	VC

This HER2-low model was developed by using 90% of transfer learning from an earlier developed HER2 model, with an additional 78 annotated regions for calibration purposes. For advanced training parameters, a defined field of view and complexity level (determined by the complexity of the feature of interest) was defined for the different layers (Table 1). A total of 7000 iterations were executed to train the AI model on three WSI, with an overall training loss of 0.0015. The HER2-low model was batch-analyzed on WSI or regions of interest for the whole set of images used in this study.

### Statistical analysis

A descriptive analysis of HER2 status was conducted across patient samples over time (core biopsy, matching surgical specimen, and matching distant metastasis) and across the three different analysis modalities (conventional microscopy, visual estimation of the scanned image on the screen, and AI), with Sankey diagrams employed for a visual presentation.

Agreement in HER2 status between the modalities was evaluated using Gwett's AC1 coefficient and weighted kappa coefficient and illustrated graphically with bubble plots and Bland–Altman plots with 95% limits of agreement across each sample type (core biopsy, surgical specimen, and distant metastasis). Correlations between methods were evaluated using Spearman’s nonparametric rank correlation coefficient.

Statistical analyses were conducted using SAS/STAT® Software, Version 9.4 (SAS Institute Inc., Cary, NC, USA).

## Results

### Patients

We identified 47 patients diagnosed with invasive BC through core biopsy or cytology, who subsequently underwent surgery with curative intent. Of the 47 patients, 37 (79%) were classified as having hormone receptor-positive BC (luminal), and 10 (21%) as having triple-negative BC (TNBC). Ten patients diagnosed with BC received neoadjuvant chemotherapy before surgery (6 luminal BC and 4 TNBC). All 47 patients experienced a recurrence of their disease in the form of distant metastases. The most common site of metastasis was the liver, followed by the skeleton. In all cases, a biopsy of the metastatic site was performed, and in 2 cases, biopsies were taken from 2 metastatic sites, resulting in a total of 49 biopsies from the metastases (Fig. 1).

### HER2 status assessment by digital image analysis using AI

Digital image analysis (DIA) using AI confirmed the HER2-low status in the majority of cases. Moreover, AI evaluated HER2-ultralow status with confidence (tumor cells with weak membranous staining: > score 0 < score 1 +) giving an exact percentage of tumor cells showing score 1 + . This was also the case when identifying HER2-null status (score 0 or 1 + in < 1% of tumor cells) or by evaluating the presence of some tumor cells with score 3 + in < 10% within an obvious HER2low tumor. Seven cases needed to be re-evaluated by AI because AI incorrectly included the DCIS component of the carcinoma (1 core biopsy and 6 surgical specimens).

### Comparison of HER2 status assessment by conventional light microscopy, digital pathology and artificial intelligence

#### Comparison of the three modalities in core biopsies

**Fig. 2:**
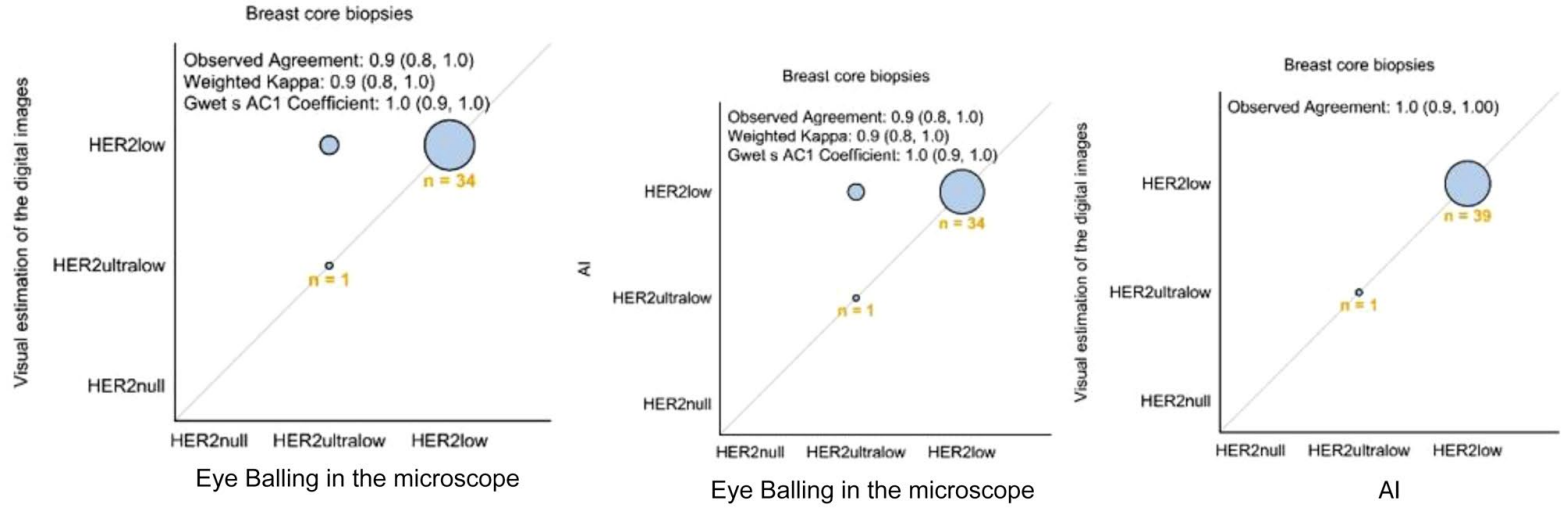
Comparison of results by the three modalities in the core biopsies. The figure illustrates the concordance between the three different assessment methods in the core biopsies

**Fig. 3:**
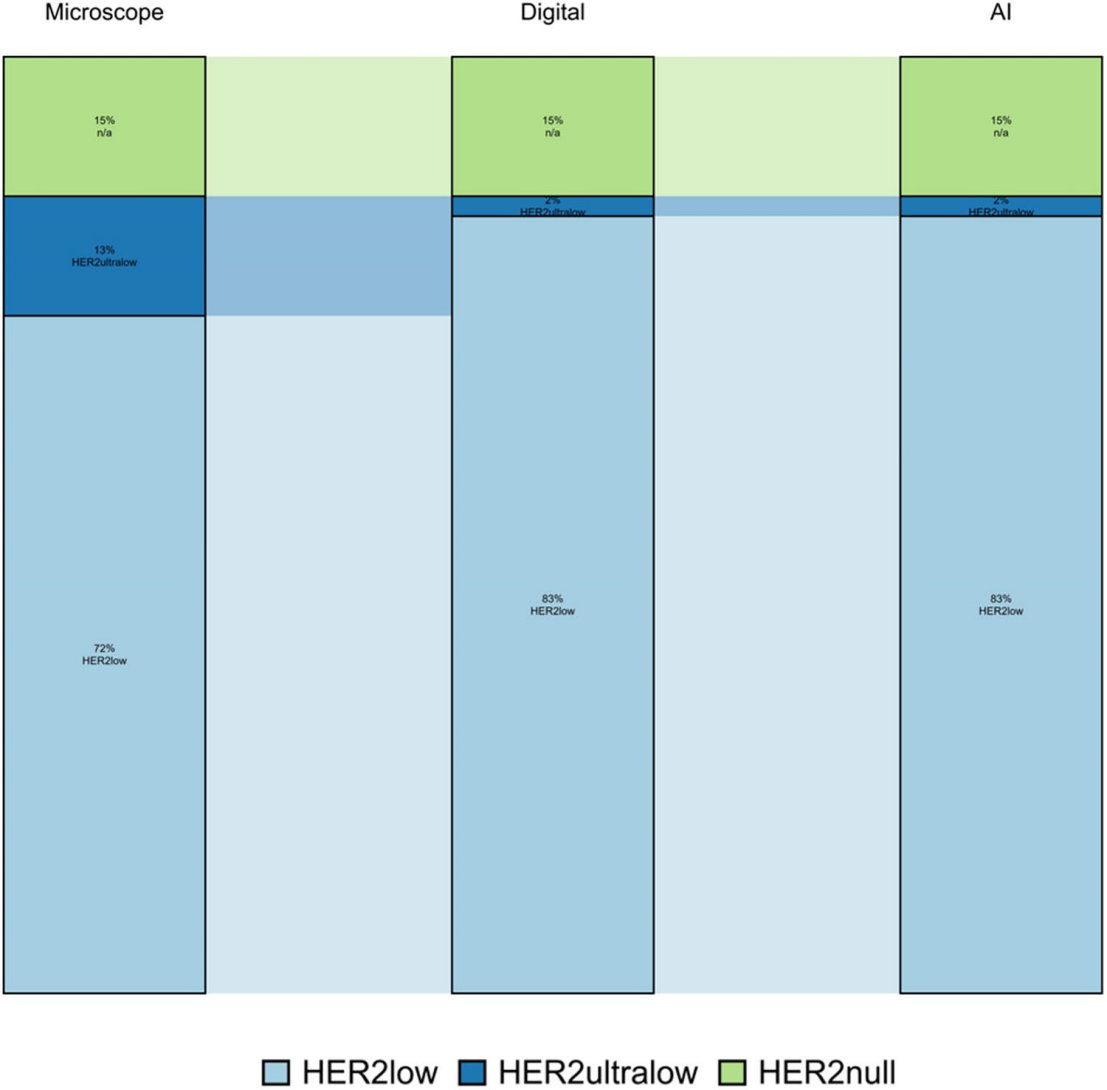
Comparison of results by the three modalities in the core biopsies. The figure illustrates the difference in results between the three different assessment methods in the core biopsies

Digital visual estimation and AI scored the same number of cases as HER2-low status, which was 10% higher compared to conventional microscopy. There was no discrepancy regarding HER2null status among the three modalities. In core biopsies, there was discordance for 5 cases, which were classified as HER2-ultralow by conventional microscopy, but HER2-low by digital visual estimation (13% discordance: 5 of 40 cases; Table 2). The observed agreement was 90% (80%, 100%) and Weighted Kappa 0.9 (0.8, 1.0) for both AI and digital estimation vs conventional microscopy. The observed agreement for visual digital estimation vs AI was 100% (90%, 100%; Figs. 2 and 3).

**Table 2:** Discordant cases among core biopsies

Patient No	Eye balling microscope	Digital image estimation eye balling on the screen	AI
Case 11	HER2ultralow	HER2low	HER2low
Case 19	HER2ultralow	HER2low	HER2low
Case 31	HER2ultralow	HER2low	HER2low
Case 32	HER2ultralow	HER2low	HER2low
Case 37	HER2ultralow	HER2low	HER2low

#### Comparison of the three modalities in surgical specimens:

Digital visual evaluation and AI classified marginally more patient samples as HER2low (51% vs 53% vs 47%) compared to microscopy. AI scored more patients as HER2-ultralow compared to microscopy and digital visual evaluation (28% vs 23% vs 19%). Consequently, more patients with HER2null status were identified using the microscopic evaluation, further highlighting that the digital image shows stronger membranous staining, and more cells could be identified with these staining patterns.

**Fig. 4:**
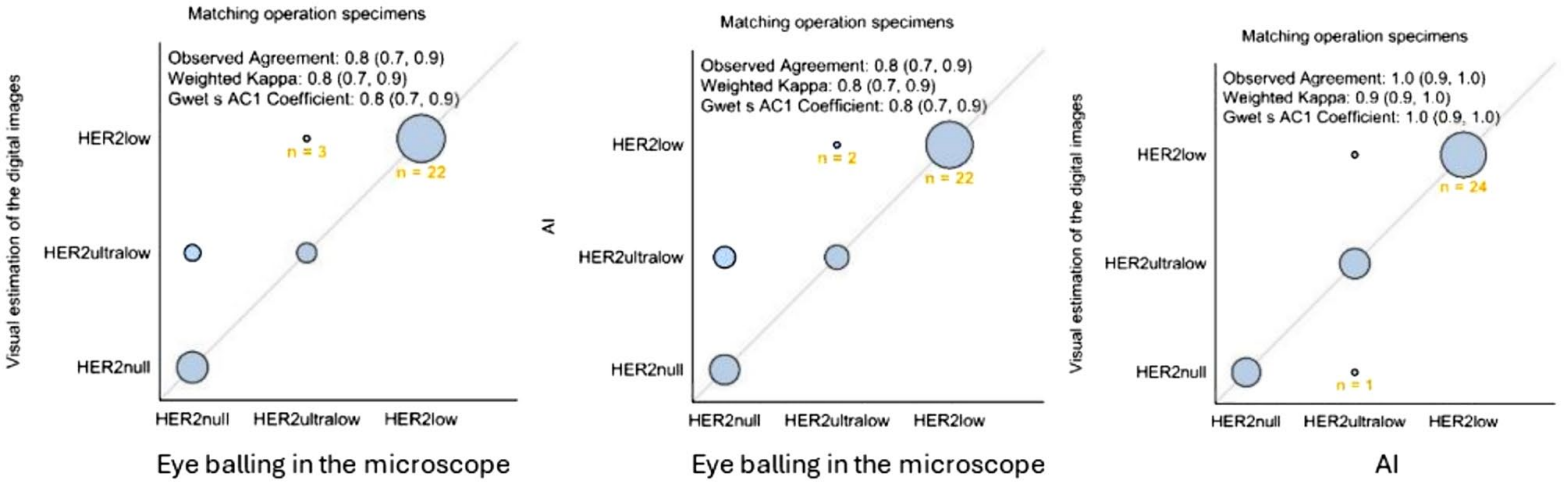
Comparison of results by the three modalities in the surgical specimens. The figure illustrates the concordance between the three different assessment methods in the surgical specimens

**Fig. 5:**
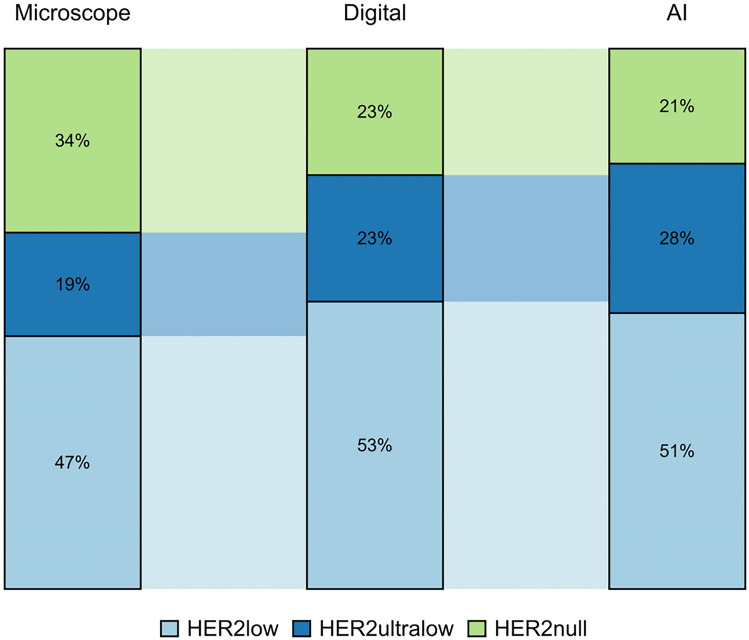
Comparison of results by the three modalities in the surgical specimens. The figure illustrates the difference in results between the three different assessment methods in the surgical specimens

In surgical specimens, there was discordance in: 10 cases (21% discordance: 10 of 47 cases) (Table 3). The observed agreement was 80% (70%, 90%) and Weighted Kappa was 0.8 (0.7, 0.9) for both AI and digital estimation vs the conventional microscope, the observed agreement digital estimation vs AI was 100% (90%, 100%) and Weighted Kappa 0.9 (0.9, 1.0; Figs. 4 and 5).

**Table 3:**
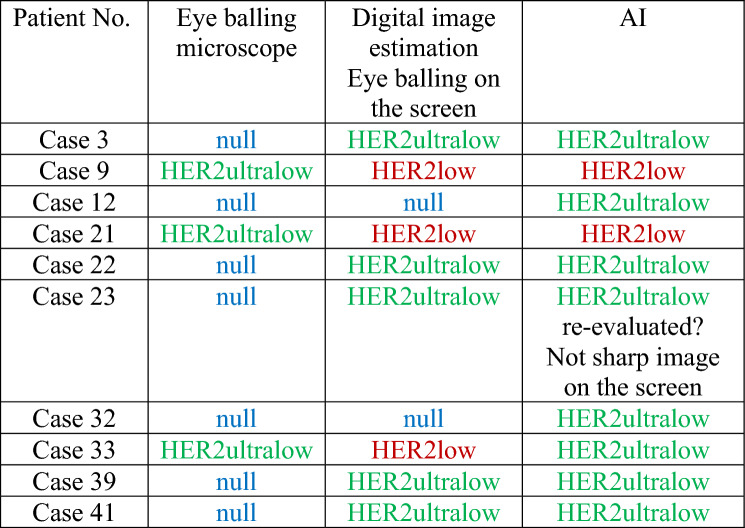
Discordant cases among surgical specimens

#### Comparison of the three modalities in the distant metastases

**Fig. 6:**
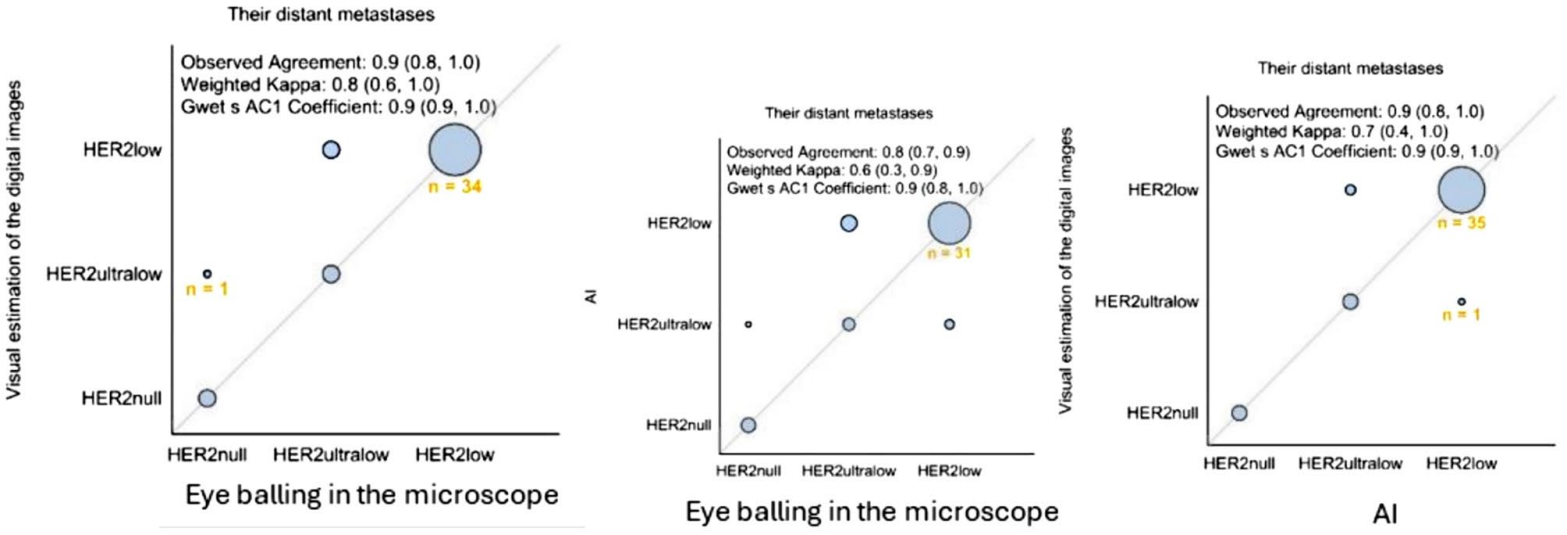
Comparison of results by the three modalities in the surgical specimens. The figure illustrates the concordance between the three different assessment methods in the matching distant metastases

**Fig. 7:**
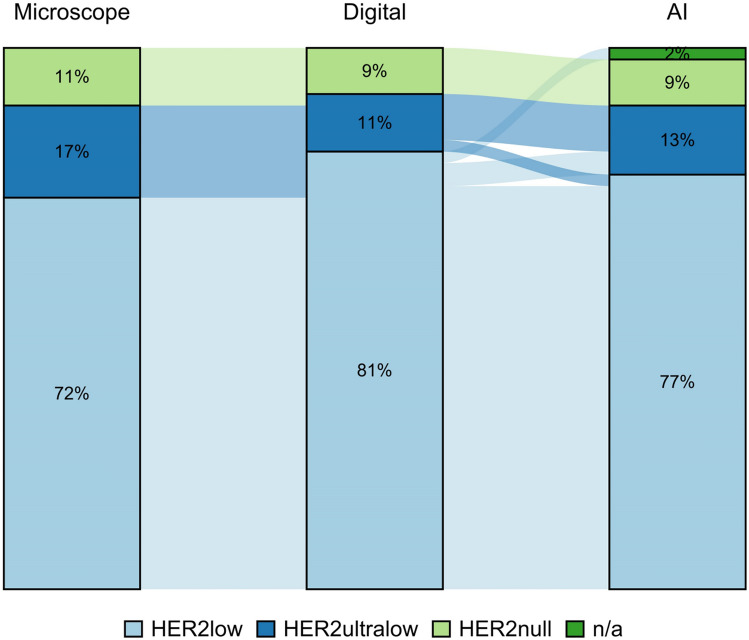
Comparison of results by the three modalities in the surgical specimens. The figure illustrates the difference in results between the three different assessment methods in the matching distant metastases

AI classified more patient samples as HER2low than digital visual evaluation (13% vs 11%). In metastatic specimens, there was discordance in: 7 cases (15% discordance: 7 of 47 cases; Table 4). The observed agreement was 90% (80%, 100%) and Weighted Kappa 0.8 (0.6, 1.0) for digital estimation vs the conventional microscope, whereas the observed agreement was 80% (70%, 90%) and Weighted Kappa 0.6 (0.3, 0.9) for AI vs conventional microscope. Moreover, the observed agreement was 90% (80%, 100%) and Weighted Kappa 0.9 (0.9, 1.0) for visual digital estimation vs AI (Figs. 6 and 7).

**Table 4:** Discordance among distant metastases

Patient No. in Excel Table	Eye balling microscope	Digital image estimation eye balling on the screen	AI
Case 3	HER2ultralow	HER2low	HER2low
Case 10	null	HER2ultralow	HER2ultralow
Case 25	HER2ultralow	HER2low	HER2low
Case 28	HER2ultralow	HER2low	HER2low
Case 29	HER2ultralow	HER2ultralow	HER2low
Case 44	HER2ultralow	HER2low	HER2low
Case 47	HER2ultralow	HER2low	HER2low

**Comparison of HER2 status in the three sample types** (core biopsy, matching surgical specimen, and matching distant metastasis).

#### Comparison of the three sample types using conventional microscopy

**Fig. 8:**
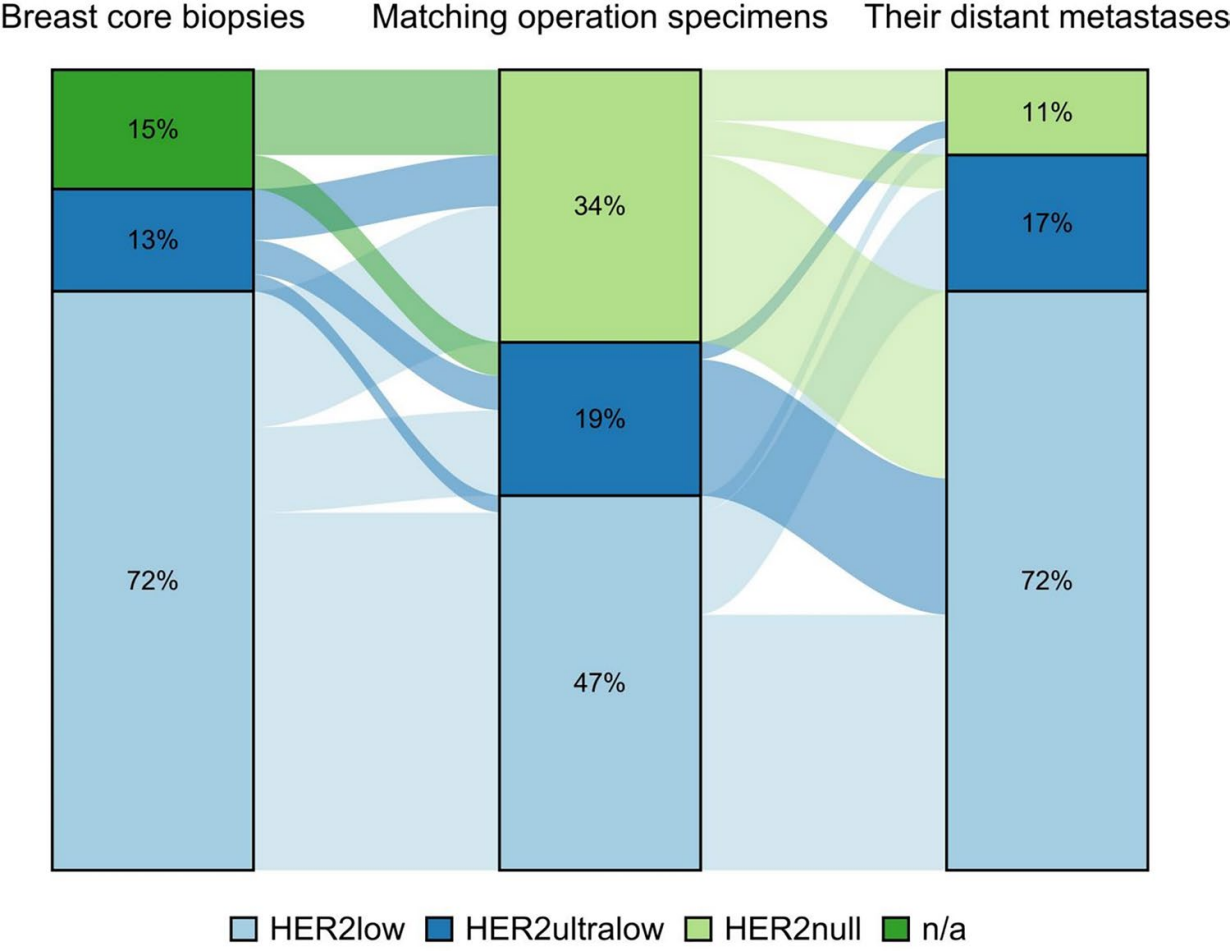
Comparing HER2 status in the three patient samples by the microscope. The figure illustrates changes in HER2 status in individual patients between different biopsy sites examined under the microscope

Using conventional microscopy, no difference was found in the number of core biopsies and matching distant metastases samples classified as HER2-low or HER2-ultralow in the three patient sample categories. None of the core biopsies were classified as HER2-null (Fig. 8).

#### Comparison of the three sample types using digital visual evaluation

**Fig. 9:**
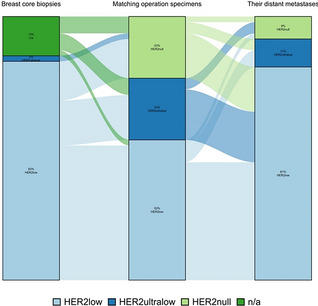
Comparing HER2 status in the three patient samples by eye balling. The figure illustrates changes in HER2 status in individual patients between different biopsy sites examined by eye balling

As with conventional microscopy, the number of samples classified as HER2-low using digital visual evaluation was similar for the core biopsies and distant metastasis samples. However, more surgical specimens and distant metastasis samples were classified as HER2-ultralow compared to core biopsies. None of the core biopsy samples were HER2-null, thereby matching the results of conventional microscopy. More HER2-low cases were identified by digital visual evaluation compared to conventional microscope (Fig. 9).

#### Comparison of the three sample types using AI

**Fig. 10:**
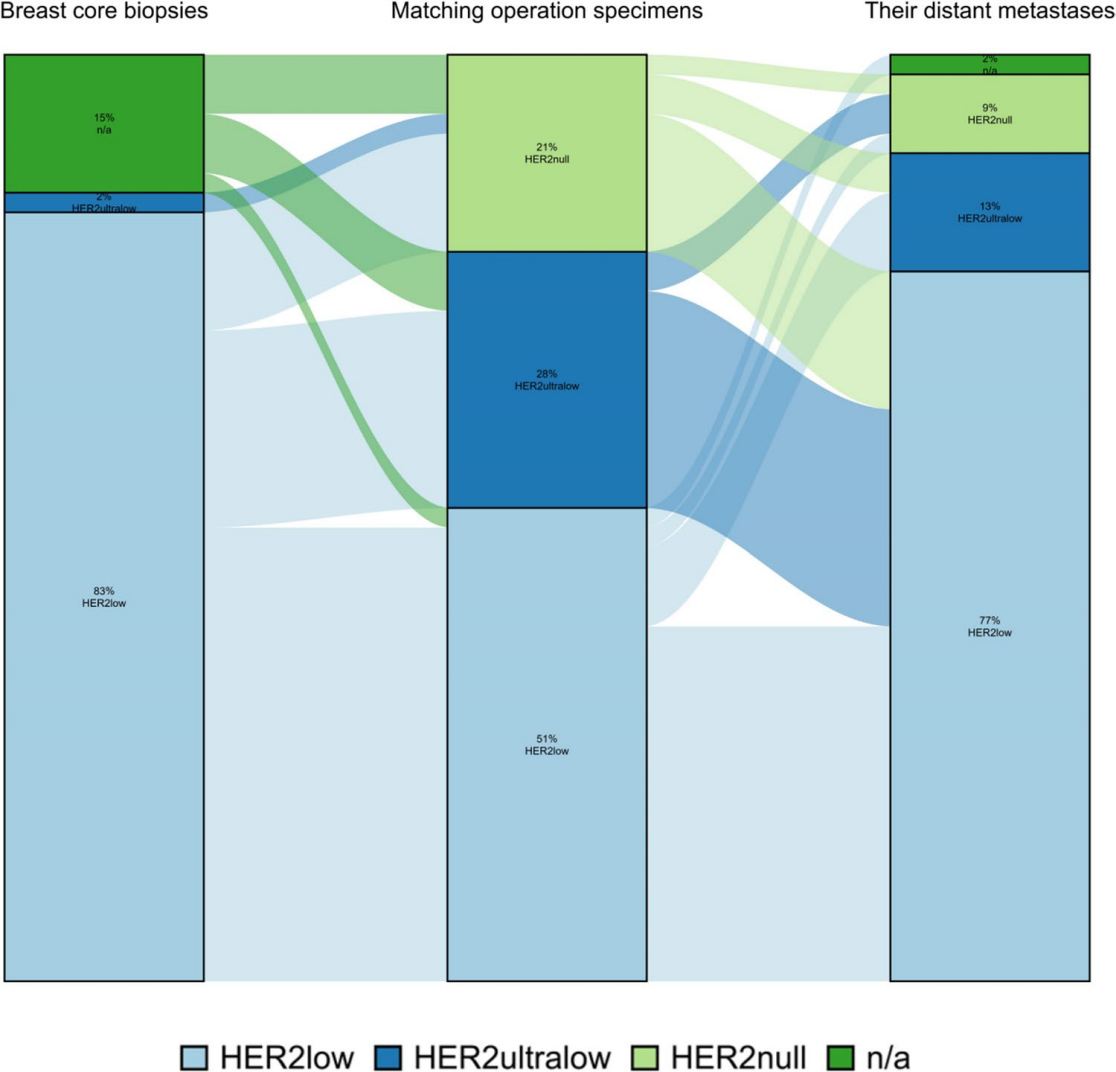
Comparing HER2 status in the three patient samples by AI. The figure illustrates changes in HER2 status in individual patients between different biopsy sites examined by AI

Consistent with the other modalities, the number of samples classified as HER2-low using AI was similar in the core biopsies and distant metastasis samples. Similar to the other modalities, the number of HER2-ultralow cases was highest in the surgical specimens, followed by the distant metastasis samples, and lowest in the core biopsies. The number of distant metastasis samples with HER2-low status was 25% higher than in surgical specimens. Fewer distant metastases were classified as HER2null compared to core biopsies and surgical specimens (Fig. 10).

### Association between treatment and changes in HER2 status

Ten patients received neoadjuvant chemotherapy (NACT), while the remaining 37 underwent surgery shortly after the diagnostic core biopsy, without receiving any antitumor treatment in the interim that could have influenced tumor biology. All patients received adjuvant treatment after surgery, which could have influenced HER2 status at the time of recurrence with metastatic disease. Therefore, we were interested in examining how HER2 status differed between the surgical specimen and the distant metastasis biopsy. We found that the most common change was an increase in HER2 expression (43%), while a smaller proportion of samples had a decrease in HER2 expression (17%; Table 5).

**Table 5:** Changes in HER2-status between the specimens

Variable	Breast core biopsies (n = 47)	Matching operation specimens (n = 47)	Their distant metastases (n = 47)	Difference matching operation specimens—Breast core biopsies	Difference their distant metastases—Matching operation specimens
AI					
n/a	7 (14.9%)	0 (0.0%)	1 (2.1%)		
HER2null	0 (0.0%)	10 (21.3%)	4 (8.5%)	Decrease17 (36.2%)	Decrease8 (17.0%)
HER2ultralow	1 (2.1%)	13 (27.7%)	6 (12.8%)	Equal23 (48.9%)	Equal19 (40.4%)
HER2low	39 (83.0%)	24 (51.1%)	36 (76.6%)	Increase7 (14.9%)	Increase20 (42.6%)

For categorical variables n (%) is presented

Effect size is estimated using the Cohen method (difference in mean/SD at first timepoint)

## Discussion

In this study, we compared three modalities—conventional microscopy, digital pathology, and an AI model—for the assessment of HER2 status and changes in HER2 expression in core biopsies, surgical specimens, and metastasis biopsies from 47 patients with advanced BC. During digital visual evaluation, the scanned images often showed a stronger membranous staining intensity than in imaging by conventional microscopy, which was consistent with previous reports. This led to higher HER2 expression scores on digital visual evaluation compared to conventional microscopy, e.g., HER2-null being scored as HER2-ultra-low and HER2-ultra-low as HER2-low. Furthermore, more tumor cells could be identified with digital visual evaluation, which resulted in a higher percentage of positively stained tumor cells. The AI model classified more patient samples as HER2-ultra-low as compared to conventional microscopy and digital visual evaluation, which can be explained by the number of tumor cells with membranous staining near the cutoff value of 10% (for HER2-low versus HER2-ultra-low status) or the cutoff value of 1% (for HER2-ultra-low versus HER2-null status). Therefore, more HER2-low cases were identified digitally and found by the AI than by conventional microscopy.

Clinically, when a patient shows radiological signs of recurrence, it is important to know how often HER2 expression changes to a lower level in the metastatic setting, particularly whether a previously HER2-low/ultra-low tumor has converted to HER2-null or 0^9^. This information is crucial in determining whether a biopsy of the metastasis is needed to confirm a prior HER2-low/ultra-low diagnosis and if the location of the metastasis is difficult to biopsy, such as in the case of brain metastases. Moreover, a patient who has undergone surgery for a HER2-low tumor is not offered adjuvant HER2-targeted therapy. However, in cases where the metastases retain HER2-low or even HER2-ultra-low status, targeted treatment with T-Dxd could offer benefits. However, according to the current practice, patients with a previous diagnosis of HER2-low or HER2-ultralow breast carcinoma may be treated with T-DXd if the site of distant metastasis is difficult to biopsy (e.g., brain metastasis).

Using all three methods, we demonstrated, that it was overwhelmingly more common for HER2 expression to be higher in the metastasis compared to the surgical specimens. In only a few cases did a HER2-low/ultralow tumor in the surgical specimen convert to HER2-null or 0, particularly in patients with a prior HER2-low tumor. Discordance between surgical specimens and metastatic biopsies has been described in other studies, which have also reported that HER2 expression most commonly increases. However, these studies compared HER2-low with HER2 0 status and did not consider HER2-ultralow as a variable [9, 12, 13]. On the other hand, studies examining HER2-positive and HER2-negative status found that it was more common for the disease to transition from being HER2-positive in the primary tumor to HER2-negative in the metastasis biopsy. This change in status has been described in up to 20% of cases, likely due to adaptation following adjuvant HER2 therapy [14]. This indicates that while the need for a new biopsy of the metastasis exists, in cases where the metastasis is in a location difficult to biopsy, it remains rather unlikely that the disease has converted to HER2-null (or 0).

One can contemplate whether the change in HER2 status concerning distant metastases may be related to the smaller biopsy size. When obtaining surgical specimens, there are larger tumor pieces available for HER2 analysis, but for distant metastases, mainly core needle biopsies are taken which represent only a random sample of the entire metastatic mass. However, we did not observe “edge artifact” in the needle biopsies by HER2 immunostaining. Decalcification of bone metastases did not modify the scores either or did not make the evaluation more difficult. Similarities in HER2-low status between core biopsies and distant metastases can be explained that both represent only a small part of the tumor (a random biopsy), not the entire tumor.

Our findings raise the question of how digital pathology and AI assessment could change the assessment of HER2 status and eligibility determination for T-Dxd compared to current standard methods. Which modality will be the gold standard? Which one should be considered authoritative? Notably, the findings of the DESTINY-04 and DESTINY-06 studies were based on evaluations conducted using conventional microscopy. The use of digital pathology varies widely across European countries and globally. Pathology departments using conventional microscopy may identify fewer advanced BCs with HER2-low status. The relatively low interobservers´ concordance in identifying HER2-ultralow status warrants the need for increased precision in HER2 assessment by digital tools using AI [15]. The recent studies have shown that AI was an accurate method for reducing the number of equivocal cases and did not affect the sensitivity of the assessment [16–20]. In our study, AI often included DCIS in the assessment, which demanded revision by excluding the areas of DCIS before the re-run. In subsequent studies, annotations should be performed in order to teach AI to exclude DCIS [21]. A discrepancy in HER2-low status using different antibodies had been reported (Ventana versus Dako´s two antibodies). Ventana´s antibody and Dako´s monoclonal antibody yields higher HER2 staining scores [22, 23].

## Conclusion

The HER2low concept causes diagnostic challenges for pathologists. We found that digital pathology with AI evaluation is a more sensitive method for HER2-low assessment of metastatic BC compared to conventional techniques (conventional microscopy and digital visual assessment) and can show HER2 expression changes across disease progression. AI evaluation provided an exact percentage of each HER2 score within seconds with very high accuracy. The Aiforia® solution highlighted specific findings and showed visual feedback, allowing users to check the results down to the pixel level. The findings emphasize the need for standardized HER2 assessment to accurately determine treatment eligibility, particularly for novel therapies like T-Dxd. Further clinical studies are needed to verify the predictive value of the scoring modalities.

## Supplementary Information

Below is the link to the electronic supplementary material.

Supplementary file1 (TIF 8786 KB)

## Data Availability

Enquiries about data availability should be directed to the authors.
