# Correction: Changes in HER2low and HER2-ultralow status in 47 advanced breast carcinoma core biopsies, matching surgical specimens, and their distant metastases assessed by conventional light microscopy, digital pathology, and artificial intelligence

**DOI:** 10.1007/s10549-025-07828-x

**Published:** 2025-09-26

**Authors:** Anikó Kovács, Leif Klint, Barbro Linderholm, Toshima Z. Parris

**Affiliations:** 1 Department of Clinical Pathology, Region Västra Götaland, Sahlgrenska University Hospital, Gothenburg, Sweden; 2 Institute of Biomedicine, Sahlgrenska Academy, University of Gothenburg, Gothenburg, Sweden; 3 Department of Oncology, Institute of Clinical Sciences, Sahlgrenska Academy, University of Gothenburg, SE-413 45 Gothenburg, Sweden; 4 Department of Oncology, Region Västra Götaland, Sahlgrenska University Hospital, Gothenburg, Sweden; 5 Sahlgrenska Center for Cancer Research, Sahlgrenska Academy, University of Gothenburg, Gothenburg, Sweden

**Correction: Breast Cancer Research and Treatment (2025) 213:397–408** 10.1007/s10549-025-07776-6

In Figs. 2, 4 and 6 of this article; the figure caption of middle figure was given as ‘Digital visual estimation’ but should have appeared as ‘Eye Balling in the microscope’.

The original article has been corrected.

