# Supplementary figures and images for: Changes in HER2low and HER2-ultralow status in 47 advanced breast carcinoma core biopsies, matching surgical specimens, and their distant metastases assessed by conventional light microscopy, digital pathology, and artificial intelligence
Source: Breast Cancer Res Treat. 2025 Jul 22;213(3):397–408. doi: 10.1007/s10549-025-07776-6 (PMC12396990; doi:10.1007/s10549-025-07776-6)

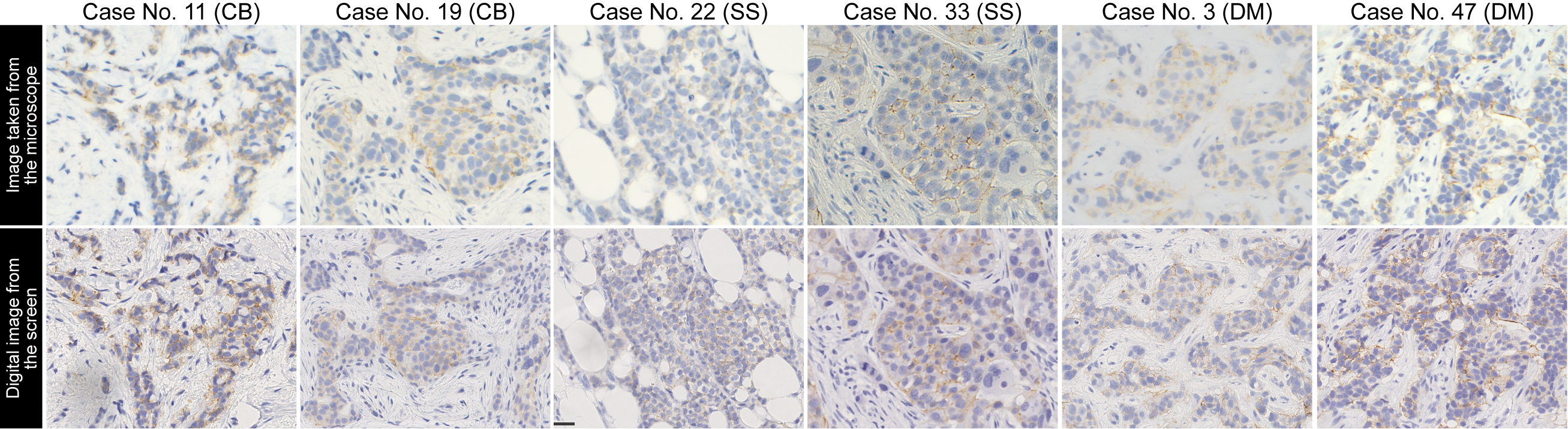

Supplement: Supplementary file 1 — Supplementary file1 (TIF 8786 KB) [file 10549_2025_7776_MOESM1_ESM.tif]
